# Sensory and Physicochemical Analysis of Meat from Bovine Breeds in Different Livestock Production Systems, Pre-Slaughter Handling Conditions, and Ageing Time

**DOI:** 10.3390/foods9020176

**Published:** 2020-02-11

**Authors:** María López-Pedrouso, Raquel Rodríguez-Vázquez, Laura Purriños, Mamen Oliván, Susana García-Torres, Miguel Ángel Sentandreu, José Manuel Lorenzo, Carlos Zapata, Daniel Franco

**Affiliations:** 1 Department of Zoology, Genetics and Physical Anthropology, University of Santiago de Compostela, 15872 Santiago de Compostela, Spain; mariadolores.lopez@usc.es (M.L.-P.); raquelmaside@gmail.com (R.R.-V.); c.zapata@usc.es (C.Z.); 2 Centro Tecnológico de la Carne de Galicia, Rúa Galicia N° 4, Parque Tecnológico de Galicia, 32900 San Cibrao das Viñas, Spain; laurapurrinos@ceteca.net (L.P.); jmlorenzo@ceteca.net (J.M.L.); 3 Servicio Regional de Investigación y Desarrollo Agroalimentario (SERIDA), Apdo. 13, 33300 Villaviciosa, Spain; mcolivan@serida.org; 4 ISPA, Avda Roma s/n, 33011 Oviedo, Spain; 5 CICYTEX (Centro de Investigaciones Científicas y Tecnológicas de Extremadura), Junta de Extremadura. Ctra. A-V, Km372, 06187 Guadajira, Spain; garsus15@hotmail.com; 6 Instituto de Agroquímica y Tecnología de Alimentos (CSIC), Avenida Agustín Escardino, 7, Paterna, 46980 Valencia, Spain; ciesen@iata.csic.es

**Keywords:** beef, principal component analysis, discriminant analysis, ageing, flavor, odor and texture

## Abstract

Different bovine breeds and production systems are used worldwide, giving rise to differences in intrinsic and extrinsic characteristics of beef. In order to meet the consumer requirements, new approaches are currently being developed to guarantee tenderness, taste, and juiciness of beef. However, the final consumer perception is complex, and it is also affected by several interrelated variables. This study aimed to evaluate the physicochemical parameters and sensory profile of three Spanish cattle breeds under different livestock production systems (extensive and intensive) and pre-slaughter handling conditions (mixing and not mixing with unfamiliar individuals at *pre-mortem* time). Meat samples from each group were also studied at different ageing times (7 and 14 days). Regarding sensory attributes, twelve panelists assessed meat samples and an exhaustive statistical analysis was carried out. The most evident and strongest effect was the breed type, allowing a great differentiation among them using principal components and discriminant analysis. The livestock production system was the second most important parameter, significantly affecting odor, flavor, and textural profile (fibrousness). It can be concluded that there were marked differences in the traits of these beef that could be modified by other factors in order to fulfill consumer tastes.

## 1. Introduction

The livestock production sector is improving the quality of products such as beef in order to meet the consumer requirements. Indeed, beef production increased by about 14% from 2013 to 2017 in Spain [1]. The development of breeding strategies and conservation management plans are based on genetic diversity of production animals. In this sense, there is a great interest in promoting the development of native bovine breeds for meat production industry. Eight autochthonous cattle breeds were involved in a promotion program due to the animal census and other aspects suitable for agriculture policy of Spain [2]. Among them, Asturiana de los Valles (AV), Retinta (RE), and Rubia Gallega (RG) are important local cattle breeds to produce meat. According to phylogenetic studies [3,4] the Spanish cattle breeds encompass two main groups: one group includes the RE and the other one contains the RG, while AV occupies an intermediate position. Carcass and meat quality from these breeds have been widely studied under different production systems [5,6,7,8,9,10,11,12] However, there are few papers which compare these breeds in terms of meat quality traits, and little is available on sensory and flavor characteristics of these three breeds, with two exceptions [8,13].

Within the broad concept of meat quality, there is a growing interest in improving the sensorial quality. Sensory acceptability is a major factor addressing the needs of the consumers and affecting their purchasing decisions [14]. Unfortunately, the sensorial profile of meat products often has a high and uncontrollable variability because of productive and technological factors and intrinsic meat characteristics. Indeed, previous studies have reported that sensory characteristics of meat are affected by both *ante-mortem* (feeding, management, and transport previous to slaughter) and *post-mortem* (ageing conditions (time and temperature), packaging, and cooking) factors, leading to significant variability [15,16,17]. In order to understand consumer responses, physiological and psychological factors have to be taken into account, something that represents a complex issue [18]. To overcome this, a descriptive sensory analysis of trained panelists can provide objective information on flavor, taste, and texture.

Within beef palatability, tenderness and odor are usually considered key factors for consumer acceptability. Specifically, the most important sensory meat attributes are related to visual appearance and in-mouth perception in terms of texture and flavor. All these quality traits could be improved to change consumer acceptance [19]. Texture is a multiparameter sensory attribute (tenderness, juiciness, smoothness, fibrousness, and coarseness), among which tenderness and juiciness are the most positively appreciated. Moreover, meat flavor is produced after a thermal process in which volatile compounds are released mainly due to lipid degradation and Maillard reactions [20]. Sensorial attributes are influenced by animal age, breed, feeding regime, pre-slaughter handling, and ageing [21,22].

Amongst these factors, breed is one of the most important, but different livestock production systems and pre-slaughter handling conditions such as transport, lairage time, slaughterhouse noises, or mixing with unfamiliar individuals are also important. All these factors may influence cattle meat quality, depending largely on animal susceptibility and breed [23,24]. In particular, pre-slaughter animal mixing is a factor that usually has negative consequences on beef quality, such as the incidence of dark cuts [23,25]. However, little is known about its influence on sensorial features and sensory quality, being difficult to find correlations with chemical composition or pH values [15].

Therefore, the objective of this study was to assess the effect of breed, production system (E = extensive vs. I = intensive), pre-slaughter handling (M = mixed vs. NM = non-mixed with unfamiliar individuals during transport and lairage stages) and ageing time (7 and 14 days) on beef physicochemical and sensorial attributes from three Spanish native breeds (AV, RE, and RG).

## 2. Materials and Methods

### 2.1. Animals and Sampling

Seventy-eight calves (24 AV, 22 RE, and 32 RG) were used in this study. AV and RG breeds are located in the north of Spain (Asturias and Galicia, respectively), while RE breed is found in the south-west of Spain (Extremadura). AV and RE calves were obtained from the experimental herd of Servicio Regional de Investigación y Desarrollo Agroalimentario (SERIDA, Villaviciosa, Asturias) and Centro de Investigaciones Científicas y Tecnológicas de Extremadura (CICYTEX, Badajoz, Extremadura), respectively. Calves from RG reared under extensive system were obtained from a family farm located in Chantada (Lugo), while those calves reared under intensive system were provided by COREN cooperative (Sarriaus, Ourense). From the total number of calves, half of the animals were reared in extensive system (E) based on natural pasture and supplementation with commercial feeding, whereas the other half only had access to commercial feeding in the finishing period (I). Calves from RG were slaughtered at 9 months, whereas AV and RE were slaughtered at 15 months of age, according to the commercial (local market and protected geographical indication (PGI) requirements) slaughter ages determined for each breed. Previous to slaughter, half of the calves from each management system (E and I) were together all time (finishing period, transport and lairage time, non-mixed treatment (NM)), whereas the other half were mixed with unfamiliar individuals from other farmers during transport and lairage stages, named mixed (M).

Calves were stunned with a captive bolt, slaughtered, and dressed according to current EU regulations (Council Directive 93/119/EC; OJ, 1993), in accredited abattoirs. Carcasses were chilled for 24 h in a conventional chamber at 2 °C (relative humidity: 98%). At this point, the m. *Longissimus thoracis et lumborum* (LTL) was extracted from the left half carcass between the 5th and the 10th ribs and cut into six steaks. The first steak was used for pH, color, and drip loss determination. The second steak was used for protein and lipid assessment. Four correlative steaks from the middle of LTL were used for shear force (Warner–Bratlzer test; *n* = 2) determination and sensorial analysis (*n* = 2) at 7 and 14 days. These latter steaks were cut with a 3.5 cm thickness, packed under vacuum conditions, and stored under refrigerated conditions (4 °C ± 1 °C) until analysis after the corresponding ageing time (7 and 14 days).

### 2.2. Physicochemical Analysis

At 24 h post-slaughter, the pH was measured at the 6th rib level using a digital portable pH meter equipped with a penetration probe. Meat color was recorded on three 10 mm diameter spots on the exposed cut surface of the LTL muscle at the 7th rib level. Color parameters (lightness, (L*); redness, (a*); yellowness, (b*)) were determined by a portable colorimeter (Konica Minolta CM-600d, Osaka, Japan) under fixed conditions (pulsed xenon arc lamp filtered to illuminant D65 lighting, 10° viewing angle geometry, and 8 mm aperture size), and the average value of the three spots was used. Chroma and Hue were calculated according to the next equations: Chroma (C* = √(a*^2^+ b*^2^)) and Hue (tan^−1^b*/a*).

The water holding capacity (WHC) measured as meat drip loss (DL) was determined in duplicate on 50 g fresh samples taken at 24 h *post-mortem* and placed in a special container (meat juice collector, Sarstedt, Germany). After aging (7 and 14 days), the steaks were frozen and stored at −20 °C for subsequent analysis of meat toughness, which was measured by the Warner–Bratzler (WB) shear test, on meat cooked by immersion in a water bath at 100 °C until an internal temperature of 70 °C was reached. After cooling, eight cores (1 cm^2^ in square cross-section) were extracted and subjected to a perpendicular cut by a WB shear blade using the TA.XT Plus instrument (Stable Micro Systems, London, UK). The maximum load (N) required for total split was recorded. Results were expressed as the mean WB shear force maximum value for each steak.

Lipid oxidation was assessed in duplicate by the 2-thiobarbituric acid (TBA) method of Salih et al. [26]. The thiobarbituric acid reactive substances (TBARS) values were calculated from the standard (tetraethoxypropane, TEP) curve and expressed as μg malondialdehyde/g meat.

Protein oxidation was assessed by measurement of carbonyl groups formed during incubation with 2,4-dinitrophenylhydrazine (DNPH) in 2N HCl, following the method described by Oliver et al. [27]. Protein concentration was calculated by spectrophotometry at 280 nm using bovine serum albumin (BSA) as standard. Protein oxidation was expressed as nmol carbonyls/g protein.

### 2.3. Sensorial Analysis

The sensory analysis was carried out according to regulation [28] to evaluate the influence of genotype, production system, pre-slaughter handling, and ageing time on organoleptic attributes of meat. A sensory panel composed of twelve panelists (7 women and 5 men, with ages ranging from 25 to 45 years) selected from the Meat Technology Centre of Galicia (Ourense, Spain) was trained for descriptive analysis according to [29]. Sensory evaluations were held in the closed individual booths according to Regulation UNE-ISO 8589:2010/A1:2014, under red light. Samples of LTL muscle were cooked in an oven at 180 °C until an internal temperature of 70 °C was reached, then they were offered to the taster in disposable plastic dishes, coded with a three-digit number drawn from a table of random numbers [30]. Water and unsalted toasted bread were used at the beginning of session and between samples to clean the palate and remove residual flavors. In order to carry out this study, quantitative descriptive analysis (QDA) was done and a randomized incomplete equilibrated blocks design was followed, where each panelist evaluated the samples that were identified with three-digit random numbers. Each panelist assessed 6 meat samples from 24 studied treatments in a single session, and a total of 12 sessions were conducted. The following textural attributes (tenderness, juiciness, fibrousness, coarseness, and smoothness), odor (overall and fat), and flavor (overall, fat, liver, and acid) were evaluated, and the intensity of each attribute was measured on a lineal, nonstructured scale from 0 (sensation not perceived) to 10 (maximum sensation).

### 2.4. Statistical Analysis

An analysis of variance (ANOVA) using the general lineal model (GLM) procedure was performed for physicochemical traits considered in the study (SPSS 23.0, Chicago, IL, USA). The model used was as follows:

Yij = μ + Bi + LPj + HPk + B × LPij + B × HPik + εijk

where Yij is the observation of dependent variables, μ is the overall mean, Bi is the effect of breed, LPj is the effect of livestock production, HPk is the effect of pre-slaughter handling, B × LPij and B × HPik were the interactions terms of livestock production and pre-slaughter handling with breed effect, and εijk is the residual random error associated with the observation.

XLSTAT 2018 (Addinsoft, New York, NY, USA) was used to analyze sensorial data. An ANOVA, with panelists and sessions considered as random effect, was conducted, and Tukey’s HSD mean separation test was used for post hoc analysis (α = 0.05). Principal component analysis (PCA) was carried out with the significantly different attributes, and it was conducted to establish the relation between the sensory attributes and the different production system, pre-slaughter handling, and ageing time of studied bovine breeds. An UPGMA (unweighted pair group with arithmetic mean) dendrogram was generated from sensorial attributes of samples using XLSTAT 2018 software.

A multivariate discriminant analysis was performed using all physicochemical and sensorial traits assessed in order to differentiate among groups of calves, hence a stepwise discriminate analysis was done. An “a priori” equal probability for a sample to be in one group independently of the group size was considered, and the criterion for the selection of variables was Wilk’s lambda (F-probabilities to enter and remove value of 0.05 and 0.10, respectively). A linear discriminant function containing an optimal subset of traits was done to determine the coefficients that maximize the differences among samples aged at 7 and 14 days.

## 3. Results

### 3.1. Effect of Breed, Livestock Production, and Pre-Slaughter Handling on Meat Physicochemical Traits

The pH, color, textural parameters, lipid oxidation, and protein oxidation of meat from calves of AV, RE, and RG are shown in Table 1. The effect interactions on pH values showed low significance level (*p* = 0.271 and *p* = 0.696 for B × LPS and B × HPS, respectively), demonstrating that the main effects can be examined independently. Additionally, the breed affected significantly (*p* < 0.0001) pH values, meanwhile there was no significant influence of livestock production system (E/I; *p* = 0.679) and pre-slaughter handling (M/MN; *p* = 0.512) on this parameter.

Regarding meat color, the interactions between breed and livestock production system and pre-slaughter handling were significantly in most cases, consequently the interpretation of the main effects is complex. For instance, in the case of L* and hue, the interactions of breed and pre-slaughter handling were not significant (*p* = 0.505 and *p* = 0.958, respectively), making it appropriate to analyze these effects independently.

The textural parameters also proved to be a highly complex situation from breed interaction analysis. However, the WHC, expressed as DL, did not show significant interactions, and the main effects could be analyzed separately. DL was only affected by breed effect, ranging from 1.41% to 1.92% for RE and AV, respectively, with no significant variation between AV and RG.

The lipid and protein oxidation status of the meat samples was assessed at different ageing times. Regarding TBARS values, breed interactions were displayed in all cases, except for interaction B x HPS, at 7 days. On the contrary, breed interactions appeared in a lower degree for protein oxidation. Protein oxidation in meat samples was affected by breed (*p* < 0.0001) and livestock system (*p* = 0.005) at 7 days.

### 3.2. Effect of Breed, Livestock Production, and Pre-Slaughter Handling on Meat Sensory Attributes

**Figure 1:**
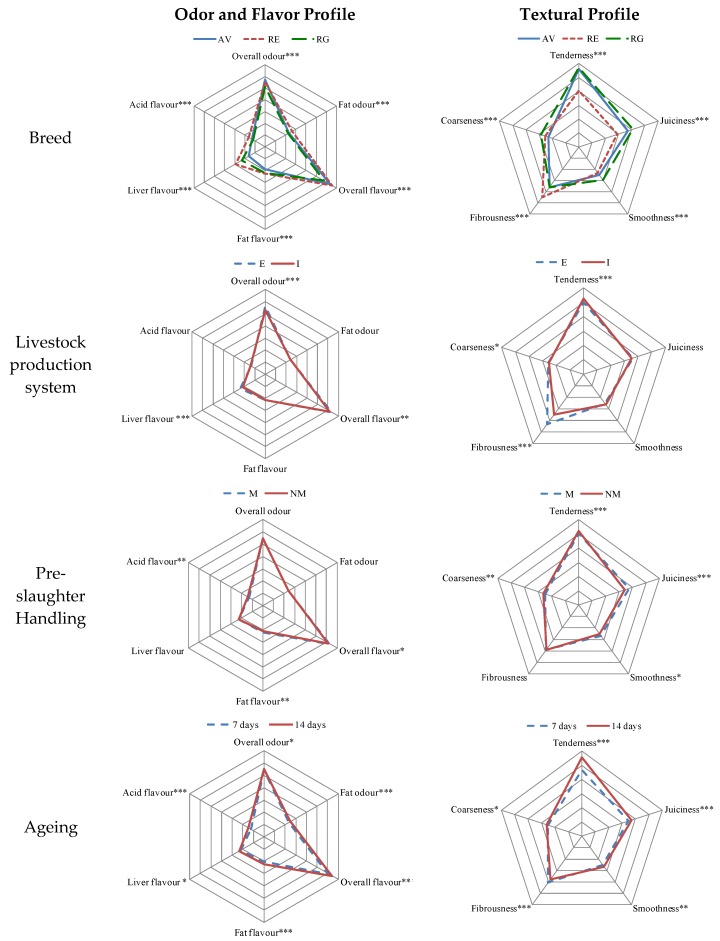
Spider plot of sensory and textural profile of Longissimus thoracis muscle for each effect: breed, livestock production system, handling pre-slaughter, and ageing. AV: Asturiana de los Valles; RE: Retinta; RG: Rubia Gallega; E = extensive, I = intensive; M = mixed, NM = non-mixed; Statistical significance: *** *p* < 0.001, ** *p* < 0.01, * *p* < 0.05.

The mean scores for the sensory and textural evaluation of beef for the effects studied in the present work (breed, livestock production system, pre-slaughter handling, and ageing) are presented in Figure 1. The results demonstrate significant differences (*p* < 0.001) among breeds for all sensory (flavor and odor) attributes. However, the significant differences in several attributes were slight (overall odor, fat odor, overall flavor, fat flavor, acid flavor, and coarseness). On the contrary, the attributes with major differences among breeds were liver flavor, tenderness, juiciness, and fibrousness. Meat from RE resulted more fibrous, less tender and juicy, meanwhile those from AV and RG proved to have more similar sensory and textural properties.

Regarding differences between extensive and intensive system, significant changes in attributes were found in overall odor, liver flavor, tenderness, and fibrousness (Figure 1). The greatest variation was displayed in the fibrousness attribute, which was more intense in samples from calves reared in an extensive system. The production system had a significant effect in tenderness, juiciness, and fibrousness (*p* < 0.001), since the greatest tenderness values were displayed in calves from an intensive system (5.23 vs. 4.97, for I and E groups, respectively), as well as the lowest value for fibrousness was coherently showed in an intensive system (3.49 vs. 4.31, for I and E groups, respectively).

Regarding pre-slaughter handling, all flavor attributes showed significant differences, as did all textural attributes, except for fibrousness (*p* > 0.05). It is remarkable that pre-slaughter mixing had a significant effect on texture, decreasing significantly the tenderness (5.21 vs. 5.03, *p* < 0.001, for NM and M groups, respectively) and increasing significantly the fibrousness (3.89 vs. 3.91, *p* < 0.001, for NM and M groups, respectively). Finally, ageing time, as expected, had the most important influence on tenderness, juiciness, and fibrousness, achieving better scores at 14 days.

### 3.3. Principal Component Discriminant Analysis for Breed Factor

**Figure 2:**
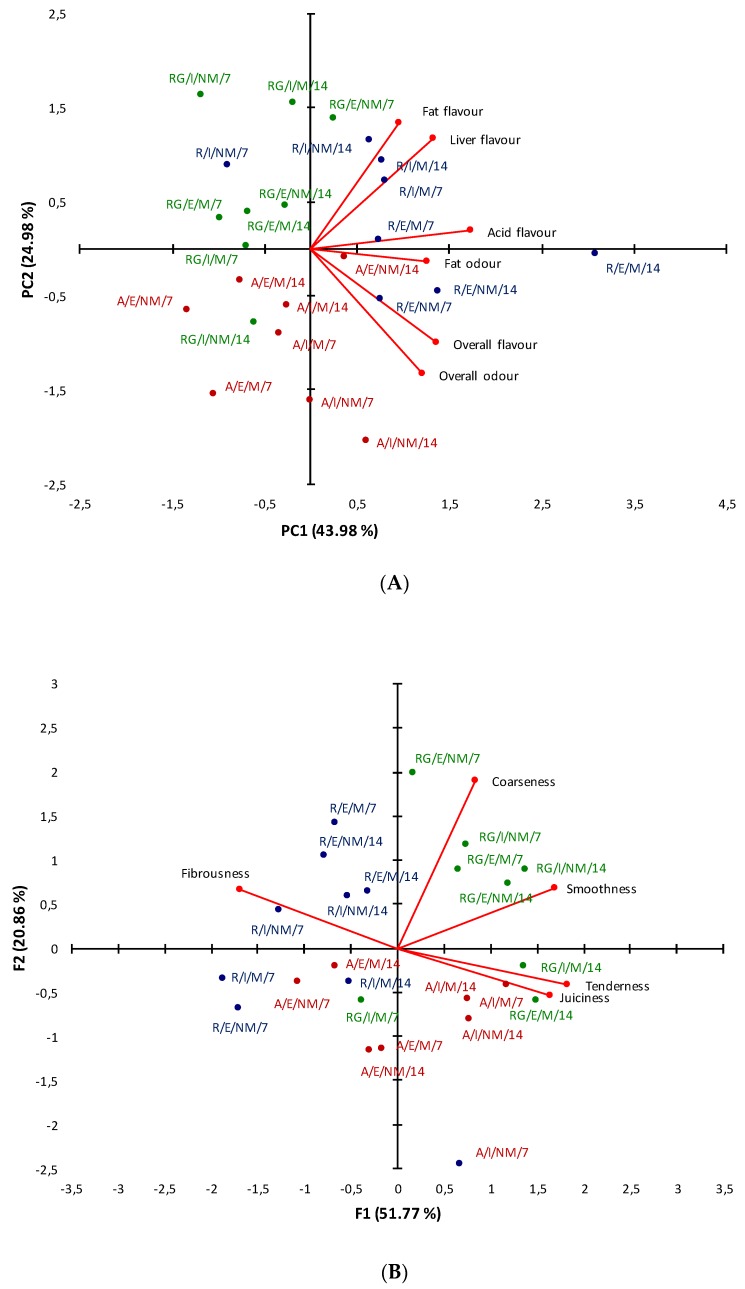
Principal component analysis (PCA) determined by principal components 1 (PC1) and 2 (PC2) of sensory and textural attributes assessed in the groups. (**A**) Flavor and odor parameters, (**B**) Textural parameters. AV: Asturiana de los Valles; RE: Retinta; RG: Rubia Gallega; E = extensive, I = intensive; M = mixed, NM = non-mixed; 7 = 7 days of ageing, 14 = 14 days of ageing.

The factor analysis aims to obtain a reduced number of principal components which would explain the variability of the samples. Sensory and textural descriptors from the 24 possible cases were analyzed to evaluate the results from a multidimensional point of view. As shown in Figure 2, a multidimensional space based on these data is reported in a bi-plot. Total variance explained for flavor and odor parameters was 68.96%. The first principal component (PC1), which explained the higher percentage of variance (43.98%), was mainly associated with flavor attributes (acid, overall, and liver in this order). Thus, AV and RG were in the left side, and most of RE in the right side. Samples from RE were well distinguished. On the other hand, the second principal component (PC2) accounted for 24.98% of the total variability and it was positively related to fat flavor and negatively correlated with overall odor. Along PC2, the samples of AV and RG were separated, with RG and AV located in the upper and lower quadrants, respectively. In addition, the most relevant aspects of RE were associated with a strong taste, placing most samples in the bi-plot right side (Figure 2A).

The second PCA was based on textural attributes, obtained from sensorial analysis (Figure 2B). The first two principal components accounted for 72.63% of total variance in data, permitting a slight separation by breeds. The projection of the variables indicated that almost all RG samples and most of the AV samples were positively characterized by coarseness, smoothness, tenderness, and juiciness, meanwhile RE samples were correlated with fibrousness. Specifically, tenderness, juiciness, and smoothness were related to meat aged at 14 days (Figure 2B).

A canonical discriminant analysis (CDA) was developed using stepwise method, and the leave-one-out cross validation was used to validate the results. A dataset composed by averages of physicochemical and sensorial parameters was subjected to the CDA, according to the breed selection. Prior to discriminant analysis, a Pearson’s correlation between sensory attributes and physicochemical parameters was done. There was a significant relationship between all textural parameters and shear force at the two ageing times explored in this study. Tenderness and juiciness were negatively correlated to shear force at 7 and 14 days (coefficients for tenderness r = −0.499 and r = −0.536, *p* < 0.01, for 7 and 14 days, respectively; coefficients for juiciness r = −0.424 and r = −0.377, *p* < 0.01, for 7 and 14 days, respectively) and the fibrousness was positively correlated to shear force at 7 and 14 days (coefficients for tenderness r = 0.324 and r = 0.536, *p* < 0.01, for 7 and 14 days, respectively).

For meat samples aged 7 days subjected to discriminant analysis, the statistical program selected the following variables: fat odor, overall flavor, liver flavor, tenderness, juiciness, fibrousness, coarseness, drip loss, L*, a*, b*, and WB-7. These variables were retained at the end of the stepwise discriminant analysis and were linearly combined to form canonical discriminant functions. Two canonical discriminant functions were used in the analysis, and the discriminant functions of classification obtained were

F1 = 0.594 [Fat_odor] + 0.211 [Overall_flavor] − 0.116 [Liver_flavor] − 0.781 [Tenderness] − 1.219 [Juiciness] + 0.673 [Fibrousness] + 0.175 [coarseness] − 0.282 [DL] + 0.510 [L*] + 1.901 [a*] − 1.156 [b*] − 0.062 [WB7]

F2 = 0.113 [Fat_odor] + 0.687 [Overall_flavor] − 0.746 [Liver_flavor] + 0.631 [Tenderness] − 0.122 [juiciness] − 0.167 [fibrousness] − 0.342 [coarseness] + 0.410 [DL] − 0.123 [L*] + 0.402 [a*] − 0.617 [b*] + 0.515 [WB7]

According to these coefficients, the parameters which mostly accounted for the segregation of F1 were color parameters (a* and b*) and juiciness, while the variables accounting for group segregation of F2 were liver and overall flavor, followed by yellowness.

For meat samples aged 14 days, the discriminant functions of classification obtained were

F1 = − 0.189 [Liver_flavor] + 0.540 [Acid_flavor] − 1.028 [Tenderness] − 0.589 [Juiciness] − 0.037 [Smoothness] + 0.563 [Fibrousness] − 0.588 [coarseness] + 0.183 [pH] + 1.809 [a*] − 0.952 [b*]

F2 = 0.868 [Liver_flavor] + 0.145 [Acid_flavor] − 0.288 [Tenderness] + 0.032 [Juiciness] + 0.580 [Smoothness] − 0.406 [Fibrousness] + 0.210 [coarseness] + 0.539 [pH] − 0.247 [a*] + 0.432 [b*]

According to these coefficients, the parameters which mostly accounted for the segregation of F1 were redness, tenderness, and yellowness. The variables accounting for group segregation of F2 were liver flavor, followed by smoothness and pH.

**Figure 3:**
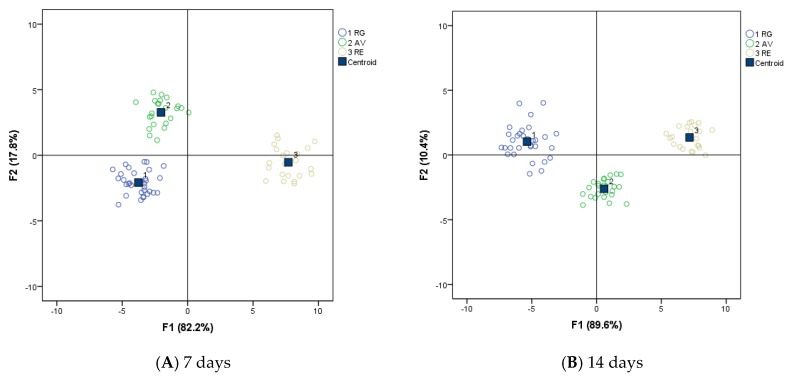
Discriminant analysis of sensory attributes affected by breed. AV: Asturiana de los Valles; R: Retinta; RG: Rubia Gallega.

All these variables were successful tools for meat discrimination of calves from different breeds. Results obtained for function F1 were plotted against results obtained from function F2 on coordinate axes for each sample, and a good discrimination among breed groups was observed (Figure 3).

For both ageing times, the function F1 explained 82.2% and 89.6%, for 7 and 14 days, respectively, while F2 explained 17.8 % and 10.4%, for 7 and 14 days, respectively, of the variance, reaching a total variance explained of 100% in both cases. A cross validation was done only for those cases in the analysis where each case was classified by the functions derived from all cases other than that case. The discriminant analysis correctly attributed each beef sample to its original group with an accuracy of 100%. Due to the number of sensory traits included in the discriminant functions, the findings of the present study suggest that these three breeds are significantly different to meet a wide range of consumer preferences.

## 4. Discussion

### 4.1. Effect of Breed, Livestock Production, and Pre-Slaughter Handling on Meat Physicochemical Traits

For all breeds, the pH values ranged from 5.48 to 5.79 and below 5.8, indicating absence of stress factors. Indeed, the most important beef quality defects are dark color, firm and dry (DFD) meat, produced from animals after a stressful period [31]. Other authors have reported variations in pH in meat by production system influence in bulls from rustic breeds [32,33]. However, in other studies those differences were not indicated [34].

The color is one of the most important meat quality traits, which is considered as the main purchasing criterion, and bright red is preferred to pale/dark red in relation to fresh meat. Variations in color traits could be due to differences in genotypes as it was previously indicated by other authors [35,36,37]. It has been reported that the extensive system contributes to change in muscle fiber types and biochemical pathway, resulting in a darker color in the majority of cases [38]. Nevertheless, our results show a clear breed interaction, resulting more complex the interpretation. Other authors found strong interactions between breed and diet regarding color parameters in beef from Aberdeen Angus and Holstein-Friesian steers, slaughtered at 19 months [39]. The production system and animal weight effects also interacted significantly in Charolais × Friesian male cattle according to Keane and Allen [40]. The combination of breed and livestock production system affected hue values, resulting that the breed effect was stronger. Our results demonstrated that the effect of livestock production system and pre-slaughter handling should be assessed within each breed.

The water holding capacity (WHC) is an important parameter in the meat quality since it plays a fundamental role in muscle organization, affecting appearance, color, tenderness, and juiciness after cooking [41,42]. The WHC was unaltered by production system, in agreement with results showed by other authors [7,43]. However, the effect of breed on drip loss was clearly important.

In this study, textural parameters were assessed by means of DL and WB tests, displaying a strong breed interaction with livestock production system and pre-slaughter handling. Additionally, the breed effect could also imply the slaughter age, as the slaughter age was breed-specific and depended on the degree of maturity and local market requirements. Tenderness variation among breeds could be explained by differences in genotype and slaughter age, as previously reported by Lucero-Borja et al. [44]. This fact also could explain the interactions, because meat quality differs with slaughter age/weight, livestock production system, and slaughter weight interactions [40]. In other studies, the effect of slaughter age on tenderness, assessed by sensorial and instrumentals methods, was contradictory, as reported by Chambaz et al. [45]. Indeed, these authors who worked with steers of four breeds with different maturity development (Angus, Simmental, Charolais, and Limousin), slaughtered with a similar intramuscular fat content (3.25%) but at different ages (381, 499, 513, and 594 days, respectively), indicated no differences in WB shear force among breed groups. However, they pointed out that meat from Angus and Limousin steers was judged by panelists significantly more tender than that of the Simmental steers, meanwhile Charolais meat was scored intermediate. Our finding does not support the previous research of Panea et al. [13], who have carried out a thorough analysis of ten European cattle breeds, resulting that AV and RE were similar in tenderness. These differences could be attributed to variations in the feeding system (Intensive), ageing time (14 days), and possible variations during the thawing and cooking process (time and/or temperature), as indicated Lepetit et al. [46], who evaluated the effect of cooking temperature/time in compression stress of several muscles in the range 20–80 °C. In our work, the effect of livestock production system and pre-slaughter handling on this parameter should be analyzed within each breed due to the interactions.

Another important consideration is the susceptibility of meat proteins to oxidative reactions, leading to a potential deleterious effect on meat quality. Our results showed the effect of breed on carbonyls level at 7 days, resulting beef samples from AV as the least oxidized. However, oxidation differences due to breed effect were reduced over time. Little is known about the relationship between protein oxidation and beef from different breeds, but the animal age is an important factor which produces an increase in carbonyl content [47]. Traore et al. [48] hypothesize about the potential relation between drip loss and protein oxidation in *longissimus thoracis* from pig, suggesting to us that both parameters strongly affected by breed effect could be related.

### 4.2. Effect of Breed, Livestock Production, and Pre-Slaughter Handling on Meat Sensory Attributes

Variations in sensory attributes (Figure 1) are consistent with those of Gregory et al. [49], who found that the textural parameters showed the highest differences among meat from nine parental breeds (Red Poll, Hereford, Angus, Limousin, Braunvieh, Pinzgauer, Gelbvieh, Simmental, and Charolais). Liver flavor attribute, which is considered an off-flavor, was more intense in RE than in AV or RG and it might be related to iron content in muscle [17], which has been reported higher in RE [50] than in AV and RG [7,8].

**Figure 4:**
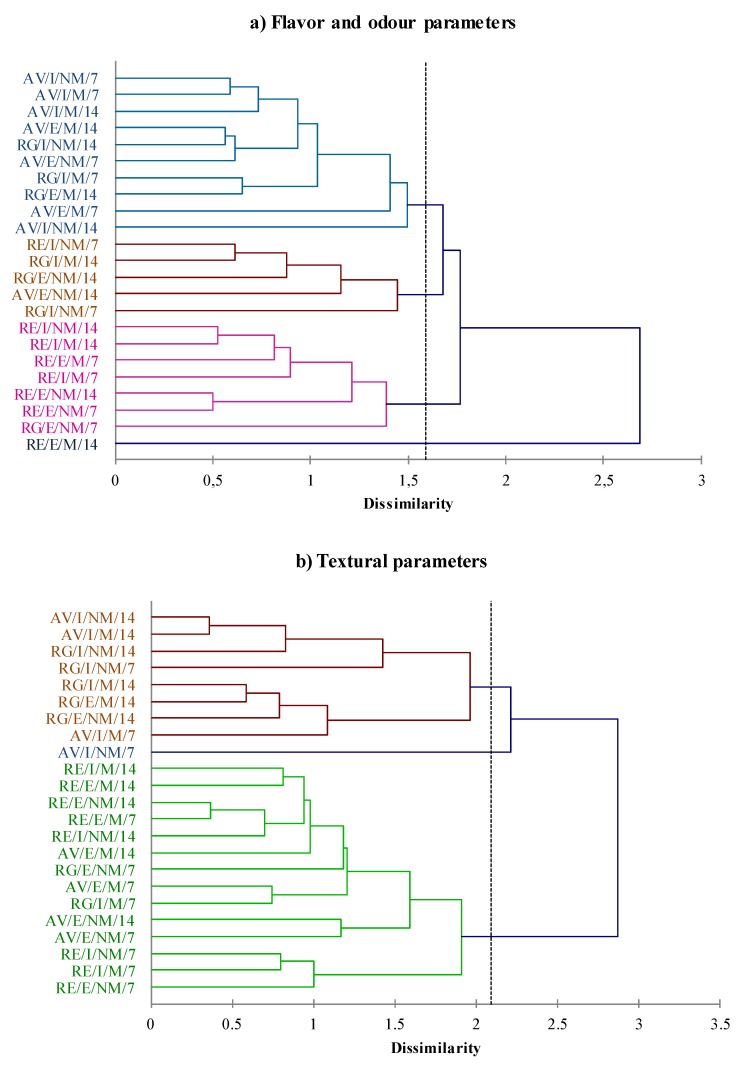
Cluster analysis using the unweighted pair group method with arithmetic mean (UPGMA) dendrogram based on flavor and odor parameters (**a**) and textural parameters (**b**). AV: Asturiana de los Valles; RE: Retinta; RG: Rubia Gallega; E = extensive, I = intensive; M = mixed, NM = non-mixed; 7 = 7 days of ageing, 14 = 14 days of ageing.

Similarity in textural parameter between AV and RG may be explained by the fact that the genetic distance between AV and RG is smaller than between AV and RE, according to phylogenetic study reported by Cañas-Álvarez et al. [3]. This hypothesis is consistent with the cluster analysis based on sensory parameters shown in Figure 4. Even though UPGMA based on flavor and odour parameters did not display a clear separation between AV and RG in differential clusters, six meat samples from RE were clustered by UPGMA analysis based on flavor and odor parameters, showing a greater differentiation between this breed and the other ones. These outcomes suggest that RE has a characteristic taste, which trained panelists clearly detected. A large amount of literature can be found attempting to unravel the influence of feeding, breed, gender, ageing, as well as food processing and cooking on beef flavor, as reviewed by Aaslyng and Meinert [22]. However, the UPGMA based on textural parameters did not provide any separation among the samples.

Sensory differences associated with extensive and intensive systems were not consistent with previous studies in which there were no differences in sensory attributes by livestock production system [32] or attributed to finishing diet [51], meanwhile Chail et al. [52] suggested that differences in sensorial attributes are due to variations in finishing diet.

Concerning eating quality, tenderness and juiciness are the most critical attributes in consumer preferences [19]. The highest tenderness values were detected from meat of calves reared in intensive systems. A possible explanation is based on differences in intramuscular fat, because it is positively correlated with palatability [53,54]. However, this result is controversial, because several studies did not report variations in tenderness between steers fed with pasture and grain [34,55]. On the contrary, other studies displayed that tenderness was higher in grain-fed calves than in grass-fed [17,56]. Another important factor that certainly contribute to meat texture and tenderness is the intramuscular collagen/connective tissue and its solubility [55,57,58]. Overall, these outcomes suggest that age, inadequate pre-slaughter management, or carcass weight could be major factors, rather than livestock production system [55,56,59]. The breed together with its typical production system under different protected geographical indications (PGI) were reported, showing differences in beef flavor, tenderness, and juiciness for three Spanish beef breeds: Bruna dels Pirineus, Avileña Negra Ibérica, and Morucha [60].

The ageing effect on sensory and textural attributes is widely known, as well as desired in order to produce necessary changes in muscle characteristics, which lead to the typical sensorial meat features, especially in beef [61,62]. As can be seen in Figure 1, ageing produced significant differences in all the attributes, being the most important those related to textural properties (tenderness, juiciness, and fibrousness). As expected, meat with longer ageing time improved the eating quality, providing superior tenderness and juiciness, as previously described Colle et al. [63]. From our results, it can be inferred that the consumption of RG meat would be recommended at short ageing times, while AV and RE meats would need a longer period of ageing to achieve their optimum acceptance by consumers.

### 4.3. Principal Component Discriminant Analysis for Breed Factor

The contribution of flavor to overall beef palatability has been demonstrated [64]. Our results obtained from PCA indicated that RE meat is very tasteful and it may be very appreciated by consumers. One of the key objectives for marketing strategies is to obtain the best sensory quality in heterogeneous products like beef. Innovations in crossbreeding, livestock feeding, and other farm aspects are currently being reviewed. In this sense, recently, a consumer study demonstrated that meat samples from RE fed organic concentrate vs. free-range were evaluated positively at the sensory level [65]. In the same line, the AV breed was analyzed by consumer study to improve their eating quality, studying ageing times and genotypes within the breed. Results from this research showed that consumer preferences changed depending on their age [8]. Unfortunately, very few sensory comparisons among these autochthonous breeds have been discussed.

In the available bibliography, similar correlations have been described between tenderness and shear force, ranging from −0.32 to −0.94 [8,66]. Apart from tenderness and juiciness, flavor and odor have been reported as the principal drivers for beef consumer acceptability [67]. In the present research, the liver flavor was positively correlated to protein oxidation at 7 days of ageing (r = 0.453, *p* < 0.01) and TBARS values with overall odor at 14 days of ageing (r = 0.336; *p* < 0.01). These finding suggest the influence of the oxidation state (protein and lipid) on the development of meat flavor and odor notes. Indeed, the development of negative flavors has been associated with beef lipid oxidation [68]. Therefore, this parameter could be negatively affecting sensorial traits of AV at 14 days. Protein oxidation has been widely researched in *post-mortem* processing meat, leading to changes in sensory attributes [69]. Overall, these facts confirm that there are hundreds of volatile compounds contributing to beef flavor and odor coming from lipid and protein breakdown products [70].

## 5. Conclusions

The present findings showed that the main quality traits of Asturiana de los Valles (AV), Retinta (RE), and Rubia Gallega (RG) were significantly different and that the effect of production system was also important to improve the beef quality. However, these great differences in physicochemical quality traits did not always lead to changes in meat sensory attributes. The results of the present study demonstrated that breed has a strong impact on fibrousness, juiciness, and tenderness, which could be used to enhance the beef texture. Although the effect of other factors such as livestock production system (including slaughter age and their possible interactions) and pre-slaughter handling on instrumental measures could play a key role in meat quality, they were less effective than breed in the sensory and textural profile. The meat ageing also modified the textural profile, which could be useful in the case of fibrous beef such as RE. According to our results, young calves of RG breed need shorter ageing than older animals of RE and AV in terms of a suitable texture. These results would be a useful tool for guaranteeing the optimal management of the meat products obtained from local breeds and to differentiate a product in the meat market. An added value of products can increase with the differentiation.

## Figures and Tables

**Table 1:** Effect of breed, livestock production system, and pre-slaughter handling on meat quality of *Longissimus thoracis et lumborum* (LTL) muscle.

	Breed				Livestock Production System			Pre-Slaughter Handling			Interactions with Breed		SEM
	AV	RE	RG	*p*-Value	E	I	*p*-Value	M	NM	*p*-Value	B × LPS	B × HPS	
**pH**	5.48 ^c^	5.79 ^a^	5.62 ^b^	<0.0001	5.63	5.65	0.678	5.64	5.64	0.512	0.271	0.696	0.019
**Color**													
Luminosity (L*)	39.38 ^b^	38.15 ^b^	41.18 ^a^	0.001	40.32	39.10	0.075	39.44	40.09	0.504	<0.0001	0.505	0.304
Redness (a*)	16.14 ^b^	22.04 ^a^	11.74 ^c^	<0.0001	14.25	18.13	<0.0001	16.65	15.36	0.101	<0.0001	<0.0001	0.211
Yellowness (b*)	11.03 ^b^	11.17 ^b^	12.54 ^a^	<0.0001	11.77	11.58	0.929	11.81	11.56	0.146	0.001	<0.0001	0.166
Croma (C*)	19.72 ^b^	26.73 ^a^	17.20 ^c^	<0.0001	19.75	21.79	0.010	21.30	20.06	0.113	<0.0001	<0.0001	0.234
Hue (h*)	36.10 ^b^	25.92 ^c^	47.08 ^a^	<0.0001	40.93	33.82	<0.0001	37.47	37.99	0.161	<0.0001	0.958	0.402
**Textural parameters**													
Drip loss (%)	1.92 ^a^	1.41 ^b^	1.89 ^a^	0.001	1.75	1.77	0.490	1.63	1.88	0.055	0.401	0.130	0.056
WB test (7 days, N)	91.7 ^b^	113.6 ^a^	66.4 ^c^	<0.0001	87.2	87.4	0.518	87.8	86.8	0.462	0.023	0.001	0.241
WB test (14 days, N)	79.5 ^b^	100.1 ^a^	58.5 ^c^	<0.0001	76.4	77.0	0.570	75.6	77.7	0.093	0.001	<0.0001	0.195
**Lipid oxidation**													
TBARS (7 days)	0.0667	0.0559	0.0687	0.671	0.0591	0.0711	0.094	0.0576	0.0710	0.251	<0.0001	0.573	0.005
TBARS (14 days)	0.1767 ^a^	0.0668 ^b^	0.0884 ^b^	0.003	0.0723	0.1551	0.001	0.0668	0.201	0.002	<0.0001	<0.0001	0.013
**Protein oxidation**													
Carbonyls (7 days)	1.48 ^b^	2.19 ^a^	1.99 ^a^	<0.0001	2.03	1.72	0.005	1.86	1.92	0.382	0.320	0.257	0.051
Carbonyls (14 days)	2.08	2.09	2.02	0.916	2.02	2.10	0.566	2.19	1.93	0.024	0.879	0.004	0.053

TBARS: thiobarbituric acid reactive substances expressed in mg malonaldehyde/kg tissue; Carbonyls expressed in nmol carbonyl/mg protein; AV: Asturiana de los Valles; RE: Retinta; RG: Rubia Gallega; WB: Warner–Bratzler; E = extensive, I = intensive; M = mixed, NM = non-mixed; B = breed; LPS = Livestock production system, HPS = pre-slaughter handling; SEM = standard error of mean; ^a–c^ Means in the same row with different letters differ significantly (*p* < 0.05).

## References

[1] FAOSTAT. http://www.fao.org/faostat/en/#data/QA/visualize.

[2] RD 2129/2008 Catálogo Oficial de Razas. https://www.mapa.gob.es/es/ganaderia/temas/zootecnia/razas-ganaderas/razas/catalogo/.

[3] Cañas-Álvarez J.J., González-Rodríguez A., Munilla S., Varona L., Díaz C., Baro J.A., Altarriba J., Molina A., Piedrafita J. (2015). Genetic diversity and divergence among Spanish beef cattle breeds assessed by a bovine high-density SNP chip. J. Anim. Sci..

[4] Mouresan E.F., González-Rodríguez A., Cañas-Álvarez J.J., Díaz C., Altarriba J., Baro J.A., Piedrafita J., Molina A., Toro M.A., Varona L. (2017). On the haplotype diversity along the genome in Spanish beef cattle populations. Livest. Sci..

[5] Piedrafita J., Quintanilla R., Sañudo C., Olleta J.L., Campo M.M., Panea B., Renand G., Turin F., Jabet S., Osoro K. (2003). Carcass quality of 10 beef cattle breeds of the Southwest of Europe in their typical production systems. Livest. Prod. Sci..

[6] Albertí P., Ripoll G., Goyache F., Lahoz F., Olleta J.L., Panea B., Sañudo C. (2005). Carcass characterisation of seven Spanish beef breeds slaughtered at two commercial weights. Meat Sci..

[7] Bispo E., Monserrat L., González L., Franco D., Moreno T. (2010). Effect of weaning status on animal performance and meat quality of Rubia Gallega calves. Meat Sci..

[8] Sierra V., Guerrero L., Fernández-Suárez V., Martínez A., Castro P., Osoro K., Rodríguez-Colunga M.J., Coto-Montes A., Oliván M. (2010). Eating quality of beef from biotypes included in the PGI “Ternera Asturiana” showing distinct physicochemical characteristics and tenderization pattern. Meat Sci..

[9] Christensen M., Ertbjerg P., Failla S., Sañudo C., Richardson R.I., Nute G.R., Olleta J.L., Panea B., Albertí P., Juárez M. (2011). Relationship between collagen characteristics, lipid content and raw and cooked texture of meat from young bulls of fifteen European breeds. Meat Sci..

[10] González L., Moreno T., Bispo E., Dugan M.E.R., Franco D. (2014). Effect of supplementing different oils: Linseed, sunflower and soybean, on animal performance, carcass characteristics, meat quality and fatty acid profile of veal from “Rubia Gallega” calves. Meat Sci..

[11] Avilés C., Martínez A.L., Domenech V., Peña F. (2015). Effect of feeding system and breed on growth performance, and carcass and meat quality traits in two continental beef breeds. Meat Sci..

[12] Horcada A., Polvillo O., Juárez M., Avilés C., Martínez A.L., Peña F. (2016). Influence of feeding system (concentrate and total mixed ration) on fatty acid profiles of beef from three lean cattle breeds. J. Food Compos. Anal..

[13] Panea B., Olleta J.L., Sañudo C., del Mar Campo M., Oliver M.A., Gispert M., Serra X., Renand G., del Carmen Oliván M., Jabet S. (2018). Effects of breed-production system on collagen, textural, and sensory traits of 10 European beef cattle breeds. J. Texture Stud..

[14] McIlveen H., Buchanan J. (2001). The impact of sensory factors on beef purchase and consumption. Nutr. Food Sci..

[15] Villarroel M., María G.A., Sañudo C., Olleta J.L., Gebresenbet G. (2003). Effect of transport time and sensorial aspects of beef meat quality. Meat Sci..

[16] Bonneau M., Lebret B. (2010). Production systems and influence on eating quality of pork. Meat Sci..

[17] Resconi V.C., Campo M.M., Font i Furnols M., Montossi F., Sañudo C. (2010). Sensory quality of beef from different finishing diets. Meat Sci..

[18] Torrico D.D., Hutchings S.C., Ha M., Bittner E.P., Fuentes S., Warner R.D., Dunshea F.R. (2018). Novel techniques to understand consumer responses towards food products: A review with a focus on meat. Meat Sci..

[19] Font-i-Furnols M., Guerrero L. (2014). Consumer preference, behavior and perception about meat and meat products: An overview. Meat Sci..

[20] Lorenzo J.M., Domínguez R. (2014). Cooking losses, lipid oxidation and formation of volatile compounds in foal meat as affected by cooking procedure. Flavour Fragr. J..

[21] Moholisa E., Hugo A., Strydom P.E., van Heerden I. (2017). The effects of animal age, feeding regime and a dietary beta-agonist on tenderness of three beef muscles. J. Sci. Food Agric..

[22] Aaslyng M.D., Meinert L. (2017). Meat flavour in pork and beef—From animal to meal. Meat Sci..

[23] Ferguson D.M., Warner R.D. (2008). Have we underestimated the impact of pre-slaughter stress on meat quality in ruminants?. Meat Sci..

[24] Muchenje V., Dzama K., Chimonyo M., Strydom P.E., Raats J.G. (2009). Relationship between pre-slaughter stress responsiveness and beef quality in three cattle breeds. Meat Sci..

[25] Weglarz A. (2011). Effect of pre-slaughter housing of different cattle categories on beef quality. Anim. Sci. Pap. Reports.

[26] Salih A.M., Smith D.M., Price J.F., Dawson L.E. (1987). Modified extraction 2-thiobarbituric acid method for measuring lipid oxidation in poultry. Poult. Sci..

[27] Oliver C.N., Ahn B.W., Moerman E.J., Goldstein S., Stadtman E.R. (1987). Age-related changes in oxidized proteins. J. Biol. Chem..

[28] ISO 13299:2016 (2016) ISO. https://www.iso.org/standard/58042.html.

[29] ISO 8586:2012 (2012) ISO. https://www.iso.org/standard/45352.html.

[30] Macfie H.J., Bratchell N., Greenhoff K., Vallis L.V. (1989). Designs To Balance the Effect of Order of Presentation and First-Order Carry-Over Effects in Hall Tests. J. Sens. Stud..

[31] Zhang X., Owens C.M., Schilling M.W. (2017). Meat: The edible flesh from mammals only or does it include poultry, fish, and seafood?. Anim. Front..

[32] Guerrero A., Sañudo C., Albertí P., Ripoll G., Campo M.M., Olleta J.L., Panea B., Khliji S., Santolaria P. (2013). Effect of production system before the finishing period on carcass, meat and fat qualities of beef. Animal.

[33] Humada M.J., Sañudo C., Serrano E. (2014). Chemical composition, vitamin E content, lipid oxidation, colour and cooking losses in meat from Tudanca bulls finished on semi-extensive or intensive systems and slaughtered at 12 or 14 months. Meat Sci..

[34] Realini C.E., Duckett S.K., Brito G.W., Dalla Rizza M., De Mattos D. (2004). Effect of pasture vs. concentrate feeding with or without antioxidants on carcass characteristics, fatty acid composition, and quality of Uruguayan beef. Meat Sci..

[35] Lanari M.C., Brewster M., Yang A., Tume R.K. (2002). Pasture and grain finishing affect the color stability of beef. J. Food Sci..

[36] Gatellier P., Mercier Y., Juin H., Renerre M. (2005). Effect of finishing mode (pasture- or mixed-diet) on lipid composition, colour stability and lipid oxidation in meat from Charolais cattle. Meat Sci..

[37] Domingo G., Iglesias A., Monserrat L., Sanchez L., Cantalapiedra J., Lorenzo J.M. (2015). Effect of crossbreeding with Limousine, Rubia Gallega and Belgium Blue on meat quality and fatty acid profile of Holstein calves. Anim. Sci. J..

[38] Dunne P.G., Monahan F.J., Moloney A.P. (2011). Current perspectives on the darker beef often reported from extensively-managed cattle: Does physical activity play a significant role?. Livest. Sci..

[39] Warren H.E., Scollan N.D., Nute G.R., Hughes S.I., Wood J.D., Richardson R.I. (2008). Effects of breed and a concentrate or grass silage diet on beef quality in cattle of 3 ages. II: Meat stability and flavour. Meat Sci..

[40] Keane M.G., Allen P. (1998). Effects of production system intensity on performance, carcass composition and meat quality of beef cattle. Livest. Prod. Sci..

[41] Hughes J.M., Oiseth S.K., Purslow P.P., Warner R.D. (2014). A structural approach to understanding the interactions between colour, water-holding capacity and tenderness. Meat Sci..

[42] Lorenzo J.M., Cittadini A., Munekata P.E., Domínguez R. (2015). Physicochemical properties of foal meat as affected by cooking methods. Meat Sci..

[43] Oliete B., Carballo J.A., Varela A., Moreno T., Monserrat L., Sánchez L. (2006). Effect of weaning status and storage time under vacuum upon physical characteristics of meat of the Rubia Gallega breed. Meat Sci..

[44] Lucero-Borja J., Pouzo L.B., de la Torre M.S., Langman L., Carduza F., Corva P.M., Santini F.J., Pavan E. (2014). Slaughter weight, sex and age effects on beef shear force and tenderness. Livest. Sci..

[45] Chambaz A., Scheeder M.R.L., Kreuzer M., Dufey P.A. (2003). Meat quality of Angus, Simmental, Charolais and Limousin steers compared at the same intramuscular fat content. Meat Sci..

[46] Lepetit J., Grajales A., Favier R. (2000). Modelling the effect of sarcomere length on collagen thermal shortening in cooked meat: Consequence on meat toughness. Meat Sci..

[47] Cho S., Kang G., Seong P.N., Park B., Kang S.M. (2015). Effect of slaughter age on the antioxidant enzyme activity, color, and oxidative stability of Korean Hanwoo (Bos taurus coreanae) cow beef. Meat Sci..

[48] Traore S., Aubry L., Gatellier P., Przybylski W., Jaworska D., Kajak-Siemaszko K., Santé-Lhoutellier V. (2012). Higher drip loss is associated with protein oxidation. Meat Sci..

[49] Gregory K.E., Cundiff L.V., Koch R.M., Dikeman M.E., Koohmaraie M. (1994). Breed effects, retained heterosis, and estimates of genetic and phenotypic parameters for carcass and meat traits of beef cattle. J. Anim. Sci..

[50] Gil M., Serra X., Gispert M., Àngels Oliver M., Sañudo C., Panea B., Olleta J.L., Campo M., Oliván M., Osoro K. (2001). The effect of breed-production systems on the myosin heavy chain 1, the biochemical characteristics and the colour variables of Longissimus thoracis from seven Spanish beef cattle breeds. Meat Sci..

[51] Moloney A.P., Mooney M.T., Troy D.J., Keane M.G. (2011). Finishing cattle at pasture at 30 months of age or indoors at 25 months of age: Effects on selected carcass and meat quality characteristics. Livest. Sci..

[52] Chail A., Legako J.F., Pitcher L.R., Griggs T.C., Ward R.E., Martini S., MacAdam J.W. (2016). Legume finishing provides beef with positive human dietary fatty acid ratios and consumer preference comparable with grain-finished beef. J. Anim. Sci..

[53] O’Quinn T.G., Brooks J.C., Polkinghorne R.J., Garmyn A.J., Johnson B.J., Starkey J.D., Rathmann R.J., Miller M.F. (2012). Consumer assessment of beef strip loin steaks of varying fat levels. J. Anim. Sci..

[54] Corbin C.H., O’Quinn T.G., Garmyn A.J., Legako J.F., Hunt M.R., Dinh T.T.N., Rathmann R.J., Brooks J.C., Miller M.F. (2015). Sensory evaluation of tender beef strip loin steaks of varying marbling levels and quality treatments. Meat Sci..

[55] French P., Riordan E.G.O., Monahan F.J., Caffrey P.J., Mooney M.T., Troy D.J., Moloney A.P. (2001). The eating quality of meat of steers fed grass and/or concentrates. Meat Sci..

[56] Kerth C.R., Braden K.W., Cox R., Kerth L.K., Rankins D.L. (2007). Carcass, sensory, fat color, and consumer acceptance characteristics of Angus-cross steers finished on ryegrass (Lolium multiflorum) forage or on a high-concentrate diet. Meat Sci..

[57] Nishimura T. (2010). The role of intramuscular connective tissue in meat texture. Anim. Sci. J..

[58] Purslow P.P. (2018). Contribution of collagen and connective tissue to cooked meat toughness; some paradigms reviewed. Meat Sci..

[59] French P., O’Riordan E.G., Monahan F.J., Caffrey P.J., Vidal M., Mooney M.T., Troy D.J., Moloney A.P. (2000). Meat quality of steers finished on autumn grass, grass silage or concentrate-based diets. Meat Sci..

[60] Serra X., Guerrero L., Guàrdia M.D., Gil M., Sañudo C., Panea B., Campo M.M., Olleta J.L., García-Cachán M.D., Piedrafita J. (2008). Eating quality of young bulls from three Spanish beef breed-production systems and its relationships with chemical and instrumental meat quality. Meat Sci..

[61] Sañudo C., Macie E.S., Olleta J.L., Villarroel M., Panea B., Albertí P. (2004). The effects of slaughter weight, breed type and ageing time on beef meat quality using two different texture devices. Meat Sci..

[62] Revilla I., Vivar-Quintana A.M. (2006). Effect of breed and ageing time on meat quality and sensory attributes of veal calves of the “Ternera de Aliste” quality label. Meat Sci..

[63] Colle M.J., Richard R.P., Killinger K.M., Bohlscheid J.C., Gray A.R., Loucks W.I., Day R.N., Cochran A.S., Nasados J.A., Doumit M.E. (2016). Influence of extended aging on beef quality characteristics and sensory perception of steaks from the biceps femoris and semimembranosus. Meat Sci..

[64] O’Quinn T.G., Legako J.F., Brooks J.C., Miller M.F. (2018). Evaluation of the contribution of tenderness, juiciness, and flavor to the overall consumer beef eating experience. Transl. Anim. Sci..

[65] García-Torres S., López-Gajardo A., Mesías F.J. (2016). Intensive vs. free-range organic beef. A preference study through consumer liking and conjoint analysis. Meat Sci..

[66] Caine W.R., Aalhus J.L., Best D.R., Dugan M.E.R., Jeremiah L.E. (2003). Relationship of texture profile analysis and Warner-Bratzler shear force with sensory characteristics of beef rib steaks. Meat Sci..

[67] Neely T.R., Lorenzen C.L., Miller R.K., Tatum J.D., Wise J.W., Taylor J.F., Buyck M.J., Reagan J.O., Savell J.W. (1998). Beef Customer Satisfaction: Role of Cut, USDA Quality Grade, and City on In-Home Consumer Ratings. J. Anim. Sci..

[68] Jiang T., Busboom J.R., Nelson M.L., Mengarelli R. (2011). Omega-3 fatty acids affected human perception of ground beef negatively. Meat Sci..

[69] Soladoye O.P., Juárez M.L., Aalhus J.L., Shand P., Estévez M. (2015). Protein oxidation in processed meat: Mechanisms and potential implications on human health. Compr. Rev. Food Sci. Food Saf..

[70] Calkins C.R., Hodgen J.M. (2007). A fresh look at meat flavor. Meat Sci..

